# A Novel Pretreatment-Free Duplex Chamber Digital PCR Detection System for the Absolute Quantitation of GMO Samples

**DOI:** 10.3390/ijms17030402

**Published:** 2016-03-18

**Authors:** Pengyu Zhu, Chenguang Wang, Kunlun Huang, Yunbo Luo, Wentao Xu

**Affiliations:** 1 Beijing Advanced Innovation Center for Food Nutrition and Human Health, College of Food Science & Nutritional Engineering, China Agricultural University, Beijing 100083, China; zhupengyu3638@163.com (P.Z.); italy10.wang@gmail.com (C.W.); huangkl009@sina.com (K.H.); lyb@cau.edu.cn (Y.L.); 2 Beijing Laboratory for Food Quality and Safety, College of Food Science and Nutritional Engineering, China Agricultural University, Beijing 100083, China

**Keywords:** GMO, digital PCR, pretreatment-free, absolute quantitative detection method

## Abstract

Digital polymerase chain reaction (PCR) has developed rapidly since it was first reported in the 1990s. However, pretreatments are often required during preparation for digital PCR, which can increase operation error. The single-plex amplification of both the target and reference genes may cause uncertainties due to the different reaction volumes and the matrix effect. In the current study, a quantitative detection system based on the pretreatment-free duplex chamber digital PCR was developed. The dynamic range, limit of quantitation (LOQ), sensitivity and specificity were evaluated taking the GA21 event as the experimental object. Moreover, to determine the factors that may influence the stability of the duplex system, we evaluated whether the pretreatments, the primary and secondary structures of the probes and the SNP effect influence the detection. The results showed that the LOQ was 0.5% and the sensitivity was 0.1%. We also found that genome digestion and single nucleotide polymorphism (SNP) sites affect the detection results, whereas the unspecific hybridization within different probes had little side effect. This indicated that the detection system was suited for both chamber-based and droplet-based digital PCR. In conclusion, we have provided a simple and flexible way of achieving absolute quantitation for genetically modified organism (GMO) genome samples using commercial digital PCR detection systems.

## 1. Introduction

Polymerase chain reaction (PCR) is considered one of the most popular detection tools used for molecular diagnosis and commercial detection. Different PCR-based methodologies have been developed and are widely used, including qualitative PCR [1,2,3,4,5], quantitative PCR [6,7,8,9,10], competitive real-time PCR [11,12], *etc.* Qualitative PCR is the most widely used since it is cost-effective and easy to perform. While qualitative PCR can be regarded as a screening method, it is only capable of achieving a yes-or-no judgment. Quantitative PCR, also known as qPCR or real-time PCR, is considered to be the most popular method for absolute quantitation; it is the “gold standard” for absolute quantitation. However, the absolute quantitation of qPCR relies on the calibration of standard curves, which can cause inconvenience and increased operation error. As a result, the coefficient of variation (CV) of the qPCR quantitation data would be varied from person to person. Additionally, the potential PCR inhibitors can have a significant influence on qPCR [13].

One example of the limitations of the qPCR method is the absolute quantitative detection of genetically modified organisms (GMOs). Since GMOs were first commercialized in the 1990s, their planting area has risen more than 100 times [14]. However, safety issues have also caught the public’s attention, in regards to the uncertain effect on human beings. To avoid GMO pollution and protect the consumers’ right to know, different countries or organizations now require labeling laws of GMOs based on the understanding of genetically modified technologies. The forced quantitative threshold areas include the European Union (EU), Japan, Korea, and Russia. The most significant regulations belong to the EU, who declared the threshold to be 0.9% instead of the previous 1%. However, the change from 1% to 0.9% could not be achieved using the current detection methods due to the uncertain CVs in the real-time PCR [15]. For the real-time PCR analysis, the absolute quantitative detection is achieved by using separate standard curves for both the endogenes and the transgenes. Therefore, both of the results may be affected by multiple factors, leading to the under- or over-estimation of the final result. Moreover, the requirement of certified reference materials, which was regarded as the basement of the generation of standard curves, limited the use of real-time PCR.

Digital PCR (dPCR) [16] is a newly developed absolute quantitative PCR-based method that is dependent on the limited separation of the reaction volume. In dPCR reaction, the PCR volume is separated to the nanoliter (nL) or picoliter (pL) level. The majority of the partitions contains no copies of the target template, whereas others contain one or more molecules. The absolute number of the target molecules in each partition is determined using a Poisson distribution by the ratio of the positive cells within the total volume based on the Poisson distribution statistics. The dPCR has been widely used for absolute quantitation in many areas, including diagnostic samples [13,17,18,19] and studies of copy number variation [20,21]. Currently, there are two types of dPCRs commercially available: droplet-based dPCR (ddPCR) and chamber-based dPCR (cdPCR). The ddPCR relies on the generation of thousands of water-in-oil droplets and obtains a positive result using a cytometry workflow. While the cdPCR is based on partitioning the reaction system using microfluidic chambers in up to a thousand separate cells. Recently, several validated GMO detection systems based on both cdPCR [22] and ddPCR [23,24] that achieved the absolute quantitation of GMO reference samples were published. Between the two platforms of digital PCR, the cdPCR can achieve the experiment results in a real-time manner. Hence, any false-positive amplification can be easily recognized and ignored. However, pretreatment steps are always included in the cdPCR procedure. Previous studies [24] showed that the detection of GMOs via ddPCR does not require the pretreatment steps. It was also mentioned that the pretreatment steps should always be included due to the equal distribution [23]. However, in the practical detection of GMO content based on cdPCR, the pretreatment steps could cause inaccurate detection results, as well as the additional inconveniences during the operation step. Therefore, to promote practical detection system for GMO content based on digital PCR, the pretreatments should be eliminated.

In the current study, a quantitative detection method based on cdPCR without any pretreatments was developed with the evaluation in the GA21 event, which was regarded as one of most famous GMO events that has been stacked with a lot of other GMO events. Additionally, to minimize the systematic CVs occurring during operating, we incorporated the duplex detection into our system. To validate our detection method, we systematically tested the dynamic range, the limit of quantitation (LOQ), the limit of detection (LOD) and the specificity in the event of GA21. Moreover, the factors that potentially affect the detection system, including the use of pretreatments and nonspecific hybridization and single nucleotide polymorphism (SNP) sites, were also evaluated.

## 2. Results

### 2.1. Determination of Dynamic Range and Limit of Quantitation

The single-plex cdPCR detection method has good linearity when the GMO content is between 0.57% and 100% [25], indicating that this method can cover approximately three orders of magnitude. In our experiment, we tested the dynamic range and LOQ of our pretreatment-free duplex cdPCR detection system.

**Figure 1:**
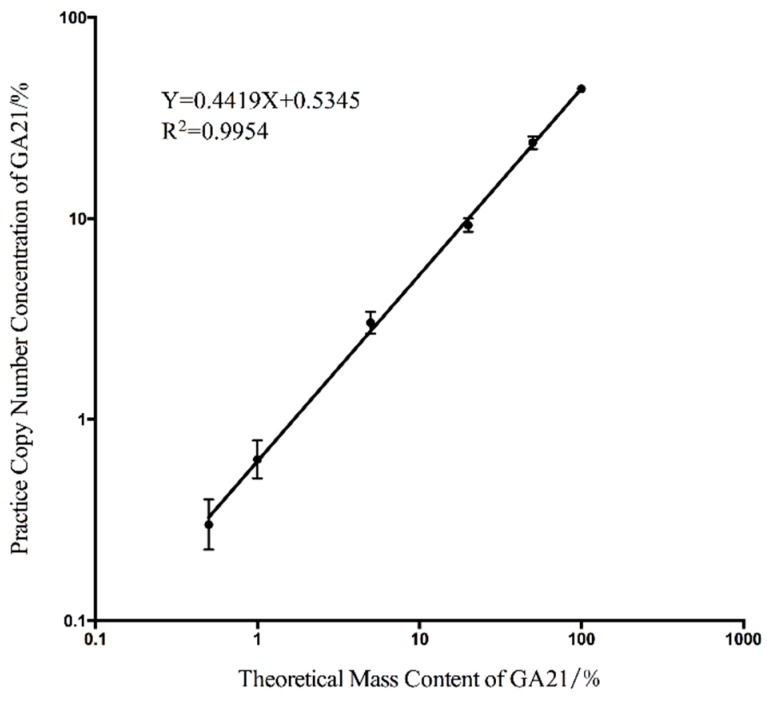
The linear regression curve between the theoretical mass content and the actual copy number concentration. In this figure, the *x*-axis means the GA21 concentration calculated by the ratio of the GA21 mass and the total mass. The *y*-axis means the GA21 content that calculated by the ratio of copy number of foreign and endogenous genes.

The ratio of the transgenic gene copy number to the endogenous genes in the transgenic maize sample was determined by the parental origin of the transgenic allele and the DNA content of the maize endosperm, which [26] was reported to vary from 36.3% to 59.4% in the maize seeds depending on the variety. According to this theory, the mass concentration cannot perfectly match the concentrations calculated by the copy number. To determine the relationship between the mass concentration and the copy number concentration and evaluate the accuracy of this detection system, the standard curve between the theoretical mass content and the actual copy number concentration was drawn (Figure 1). The good linear correlation indicated that the copy number concentration matched the mass content through a constant quantity that was determined by the slope of the standard curve. The actual copy number of each panel and the concentrations calculated using Equations (1)–(3) are listed in Table 1. The low relative standard deviation (RSD) within the range of 0.5%–100% indicated the good repeatability of our detection system within this detection area.

The final LOQ was determined using the lowest absolute copy number or copy number concentration that could be reliably detected with an acceptable precision and accuracy. For the validated EU method [27] for the quantitation of GMO content, the LOQ is recommended as the data group with an RSD value under 25%. Based on this criterion, the LOQ of our duplex cdPCR detection system was set up for a single copy of the GA21 event-specific fragment and 0.5% for the copy number concentration for the absolute quantitation. Compared with the qPCR data generated in a previous study [24], our absolute quantitative detection system performed using duplex cdPCR has a lower LOQ and better accuracy.

### 2.2. Evaluating the Sensitivity of the Duplex Digital Polymerase Chain Reaction (ddPCR)

In our experiment, we defined the LOD as the lowest GMO content percentage that can be detected reliably but not necessarily quantitatively. The GA21 samples with different mass concentrations were used to evaluate the sensitivity of our quantitative detection system. As is seen in Table 1, the positive wells could be differentiated from the negative groups. Therefore, the LOD for our detection was 0.1%, and the LOD for the absolute copy number is a single copy for the event-specific gene.

### 2.3. Evaluation of the Specificity of the ddPCR

In our study, the specificity was defined as the cross-amplification of the event-specific primers for different GMO events. In this part, the oligonucleotides of Adh-135-F/R/P and GA21-F/R/P were selected. The results of the verification of the different event-specific primers and probes showed that GA21 event-specific primers and probes could only amplify the target sequences of the GA21 event, and it was perfectly matched with the theoretical result published in the validated EU method. This indicates that the specificity of the duplex cdPCR detection system is the same as the validation result achieved using qPCR.

### 2.4. Evaluation of the Influence of the Pretreatment

**Figure 2:**
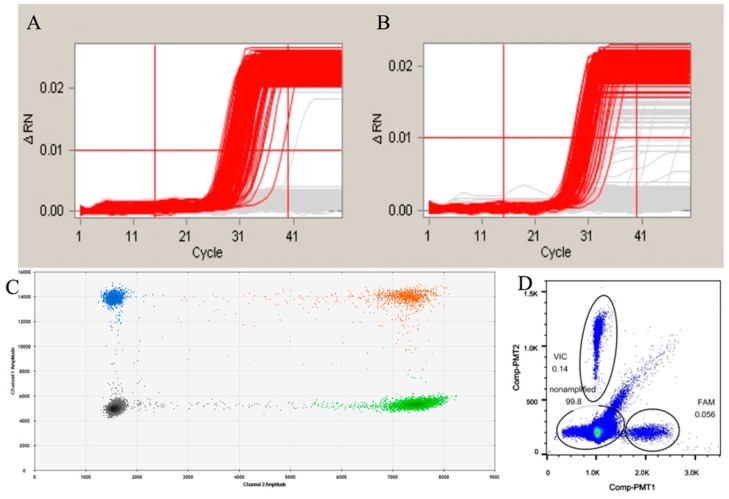
The evaluation of the digestion of the genomic DNA. (**A**,**B**) are the amplification curves generated by chamber-based digital Polymerase Chain Reaction for undigested and digested genomic DNA, respectively. The red lines mean the positive amplification curves, while the grey lines mean negative curves; (**C**,**D**) are the amplification hot maps of digested genomic DNA samples of droplet digital Polymerase Chain Reaction by the Bio-Rad and RainDrop platform, respectively. For the different colors in C, the black means negative for both FAM and VIC, the blue and green mean positive only for FAM or VIC, the orange mean positive for both FAM and VIC. The droplets in the circle of (**D**) means the positive droplets.

To evaluate the effect of the pretreatment on the final quantitative detection result, we performed quantitative analysis of genomic samples with or without the pretreatment. The cdPCR and ddPCR systems were both evaluated in this part of the experiment. The final detection result is shown in Table 2 and Figure 2. The results indicated that the pretreatments had a side effect on the final result, leading to a lower value in the ddPCR and poorer amplification curves in the cdPCR.

### 2.5. The Effect of the Nonspecific Hybridization Bases of the Probes

**Figure 3:**
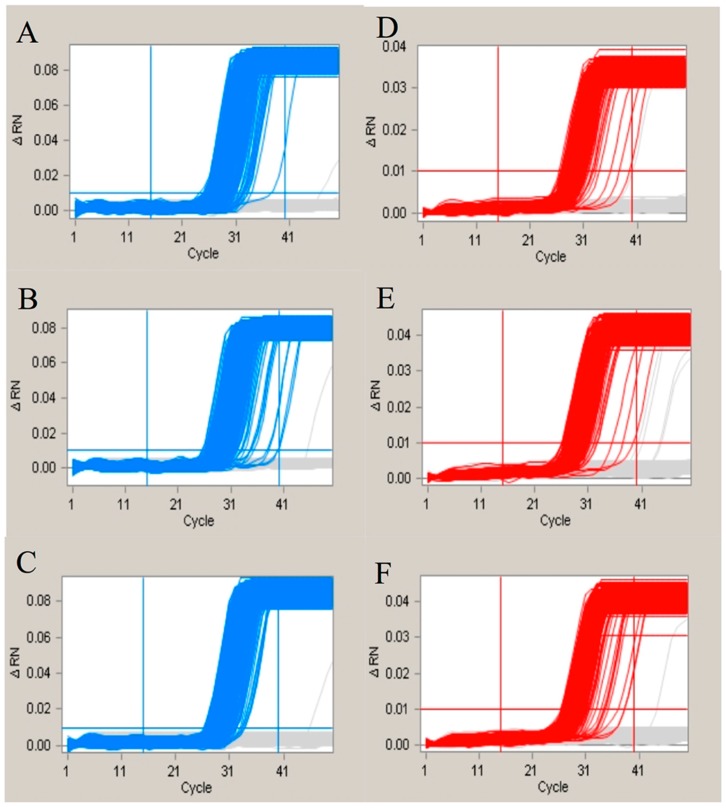
The amplification curves for evaluation of the nonspecific hybridization. The (**A**–**C**) represented the amplification curves generated by the 135 bp fragment of Adh1 gene combined with no primer, GA21-P-4 and GA21-P-5; The (**D**–**F**) represented the amplification curves generated by the GA21 event-specific gene with no primer, A135-P-4 and A135-P-5. The red lines mean the positive amplification curves, while the grey lines mean negative curves.

To evaluate the effects of nonspecific hybridization within the assays of GA21 event-specific genes and endosgenes, we designed nonspecific oligonucleotides based on the original probe sequences. The nonspecific hybridization sites and the ΔG are listed in Table 3. Based on the amplification results (Figure 3), the nonspecific hybridization with the probes showed no difference compared with the control group. We concluded that the detection system is flexible and is not affected by the sequences of the primers and probes.

### 2.6. The Influence of the Single Nucleotide Polymorphism (SNP) Sites

**Figure 4:**
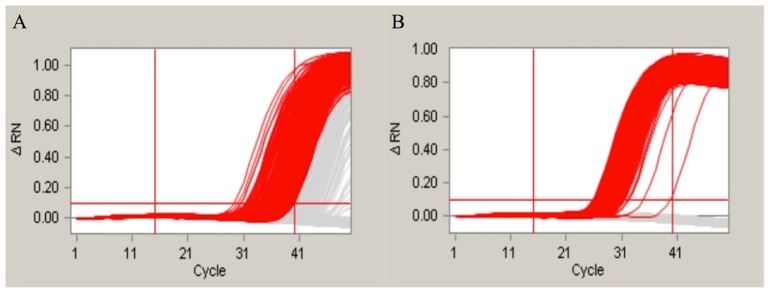
The amplification curves of 70 bp fragment of Adh1 gene; (**A**) represented the amplification curves generated by the original primers with one SNP site; and (**B**) represented the amplification curves generated by the modified primers with no SNP site.

**Figure 5:**
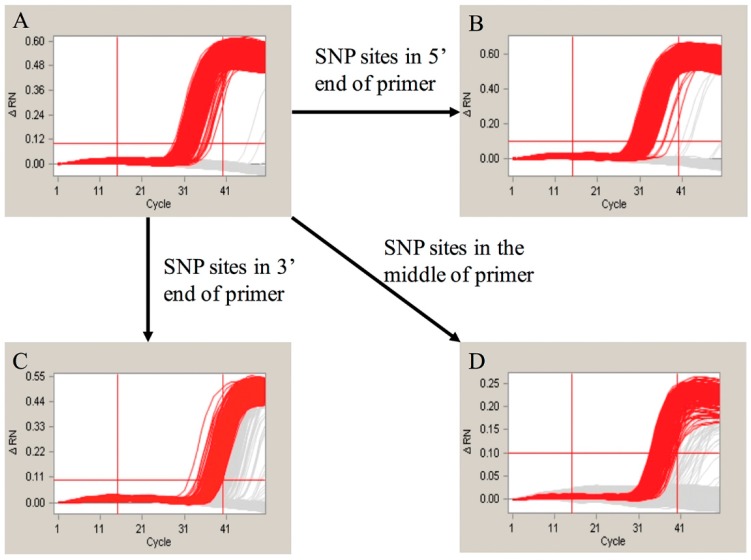
The evaluation of effect of SNP sites; (**A**) is the amplification curves of the primer without SNP site. The (**B**–**D**) represented the amplification curves for the SNP sites occurred in the 5′, 3′ and middle of Adh1-135F primer.

One SNP site located on the reverse primer for the Adh1 gene was identified in an earlier report [28]. This SNP site was detected in the amplification product of the 70-bp Adh1 gene fragment using our detection system described in this paper. To further evaluate in detail how the SNP sites affect the amplification, we designed different primers for the SNP experiment. We first altered the SNP located within the reverse primer of the 70-bp Adh1 gene fragment. The amplification product (Figure 4) supported our hypothesis that the SNP sites influence the amplification results. Moreover, we manually altered the bases in a different portion of the forward primer of the 135-bp Adh1 gene fragment to introduce SNPs. The loci of these SNPs in the Adh1 gene are shown in Figure S1. The amplification results are shown in Figure 5. The amplification results showed that the SNP sites at the 5′ end had little effect on the final result, whereas the ones located in the middle or at the 3′ end led to significantly different results compared with the control groups.

## 3. Discussion

Previous studies have shown that pretreatments, including digestion by restriction enzymes [20,21,22,24] or heat treatments [23], are needed prior to dPCR. Following these pretreatments, the genome is cut into the small linear fragments, whereas the untreated DNA remains supercoiled, which can be more difficult to separate into each reaction cell. However, pretreatments may introduce contaminants and cause an inaccuracy in the final results. For our previous study, a qualitative screening detection system based on the digital PCR without the pretreatments for the detection of GMO content has been developed [29]. The digital PCR reaction condition has been optimized to avoid the unbalanced distribution of genome samples caused by the independence of pretreatments. However, as the digital PCR was regarded as an absolute quantitative detection tool, the potential elements that might influence the absolute quantitative detection have been evaluated in our study.

To evaluate whether the digestion of the genome samples might influence the final result in both commercial dPCR systems, we adopted our detection protocol for ddPCR systems of Bio-Rad QX100 and the Raindrop^®^ droplet digital PCR. According to the detection result for the cdPCR, the amplification curves of the undigested DNA were neater than those of the digested group (Figure 2A,B). This indicates that the fragmentation of the genome may affect the amplification efficiency possibly due to random annealing of the target sequence. The Raindrop^®^ digital PCR system is a new commercial ddPCR system that can generate the smallest droplets among all of the commercial dPCR systems that function at the pL level. As the result of the ddPCR (Figure 2C and Table 2), the detected result for the digested genome was lower than that of the undigested group, which is closer to the result observed for the cdPCR. We believe that this may be due to the recycling procedure of the enzyme-cutting products. The recycling procedure was performed using spin columns, which could lead to the unbalanced recovery of oligonucleotides of different lengths. This result was further confirmed by the ddPCR data using the Bio-Rad platform (Figure 2D and Table 2). Based on the results of the quantitative detection system used in this study, the pretreatment prior to the digital PCR had an effect on the final results. Therefore, the pretreatment-free detection using cdPCR performed in our assay is essential for achieving accurate results.

To address the concern that different combinations of endogenes and event-specific genes could generate different results, we hypothesized that the final result of the duplex amplification via cdPCR might be influenced by the effect of the primary and secondary structures of the probes. We designed different primers in which several bases were altered according to the sequence of the other probe in the duplex dPCR. The sequence of the primers and corresponding probes, as well as the ΔG for the hetero-hairpin within the probes and modified primers, are shown in Table 3. According to previous studies [30,31], multiplex real-time PCR may influence the amplification efficiency of each pair of primers and probes. Therefore, we firstly used real-time PCR to determine the different amplification efficiencies between the single-plex PCR and the duplex PCR, in addition to the duplex PCR with different numbers of nonspecific hybridization bases. The real-time data for both the single-plex and duplex real-time PCRs are shown in Table S1. The amplification efficiency and linear correlation coefficient (*R*^2^) for both the single-plex and duplex PCR indicates that the amplification efficiency was not influenced by the primers and probes for the duplex PCR. Moreover, in regards to the evaluation of the nonspecific hybridization bases, different numbers of hybridization bases were manually inserted into the long primer sequences of the probes. The same results were achieved (Table S1), showing that the nonspecific hybridization did not affect the PCR amplification efficiency. This shows that duplex real-time PCR is a convenient method for using directly combined single-plex primers and probes. An evaluation of the cdPCR was then performed to determine whether the nonspecific hybridization affected the amplification in an extremely small reaction volume. The amplification curves of all of the wells for the different panels are shown in Figure 3. The results indicated that the addition of the nonspecific oligonucleotides had little influence on the duplex PCR reaction used for the cdPCR detection. Therefore, we believe that several nonspecific hybridization bases between the different probes could improve the amplification for a single pair of primers and probes, as it may decrease the synthesis of secondary structures inside each pair of primers. We have evaluated up to five nonspecific hybridization bases, and no amplification inhibition was observed. This means that the duplex amplification used for the cdPCR detection is a flexible method, as each pair of the primers and probes showed good amplification ability.

Previous studies have shown that one single SNP site in the Adh1 reference gene of GA21 existed in the EU standard primers that amplify a 70-base pair region. To evaluate whether this SNP site can influence the final results of real-time PCR and the duplex cdPCR in our study, we performed both of the methodologies. By the evaluation of the real-time PCR, the conclusion was drawn that the SNP sites have a slight influence on the amplification and standard curves of the single-plex real-time PCR. The standard curves and amplification efficiency showed differences compared with the theoretical values (Table S2). However, the difference between the SNP group and the control group was not significant that could be hardly noticed during the daily detection. This suggested that real-time PCR is a poor method for identifying SNP sites. For the cdPCR, both the single-plex and duplex detection showed distinct results between the SNP group and the normal control group. In our study, we modified the SNP site of the original primers by altering the SNP base from an A to a G to evaluate the effect of the SNP site. Based on the results (Figure 4), the SNP group showed very different amplification curves compared with the group without the SNP in both the single-plex and duplex cdPCR. This means that the modification of a single base in the primers can significantly influence the final amplification curves. Moreover, to evaluate the differences caused by different mutation sites, we manually modified the primer bases at the 5′ and 3′ ends and in the middle of the sequence. The amplification results (Figure 5) showed that the mutations at the 3′ end and in the middle had the greatest effect, whereas at the 5′ end, no significant changes were observed. This detection result fit the theory that the bases near the 3′ end of the primers have a greater suppressing effect on the level of amplification. In conclusion, our duplex digital PCR system provides a more accurate and flexible method for the detection of potential SNPs.

Our detection system based on the pretreatment-free duplex detection system developed in our article can achieve quantitative detection at levels as low as 0.5% of the mass content, with a detection limit of 0.1% that is lower than the results achieved using real-time PCR. The duplex cdPCR detection system described in this paper provides a convenient method that does not require further optimization of the duplex primers. Moreover, it is capable of directly identifying SNP sites, providing an efficient SNP detection method.

## 4. Materials and Methods

### 4.1. Transgenic Materials

The phytocide-resistant maize, GA21, was kindly provided by the Monsanto Company (St. Louis, MO, USA). The non-GM maize was stored in our lab.

### 4.2. Preparation of the Mixed Samples

Prior to extraction, the seed samples were ground using MM430 mixer (Retsch, Duesseldorf, Germany). The grinding step consisted of 10 cycles of shaking for 10 s with 1 min intervals. This step is used to protect the genome from heat injury. After grinding the seed samples, the mixed samples containing different GMO contents were mixed. In this assay, the experimental samples were set at 100%, 50%, 20%, 5%, 1%, 0.5% and 0.1%. The mixed samples were then placed in a Dynamic CM-200 mixer (Retsch, Germany) overnight for additional shaking to equally distribute the samples.

### 4.3. Extraction of the Genome

The genomic DNA was extracted from 50 µg of the powdered sample using a modified CTAB method [32]. The isolated genome was diluted with 60 µL of ddH_2_O. The concentration and purity were determined using a NanoDrop N2000 (Thermo Fisher Scientific Inc, Wilmington, DE, USA) at both OD260 and OD280. For the further digital PCR experiments, the final concentration of each samples was diluted to 50 ng/µL by adding the addition ddH_2_O.

### 4.4. Pretreatment of the DNA Samples

Restriction endonuclease treatment of the genomic samples was used to evaluate the side effects of the pretreatments on the final result. A 50 µL reaction volume was prepared, containing 5 µL of 10× buffer 2.1 (New England Biolabs, Beijing, China), 20 U of *HindIII* restriction endonuclease (New England Biolabs, Beijing, China), 2500 ng of the genome sample and ddH_2_O up to 50 µL. The reaction was performed at 37 °C for 2 h. Following digestion, the DNA was isolated using a QIAquick PCR Purification Kit (Qiagen, Duesseldorf, Germany).

### 4.5. Primers and Probes

The event-specific primers and probes were chosen from a validated European Union detection method. The Adh1 gene (GenBank: X04050.1) was used as the endogenous gene for the maize. Both the event-specific and the endogenous genes have a unique copy number in the target genome. The primes and probes were then blasted according to the sequences. The probes were labeled with the fluorescent FAM or VIC reporter dyes on the 5′ end. Additionally, the common TAMRA quencher was replaced with the Black Hole Quencher 1 (BHQ-1) in our study, as a lower fluorescent baseline was observed. All of the primers and probes were synthesized by Invitrogen (Life Technologies, Wilmington, DE, USA). The sequences of the primers and probes are listed in Table 4.

### 4.6. The Digital PCR

#### 4.6.1. The Chamber-Based Digital PCR

The digital PCR was achieved using a BioMark System (Fluidigm, South San Francisco, CA, USA) with the 48.770 digital array (Fluidigm). This digital array had 48 individual panels, which contained 770 individual partitions. A 4 µL reaction mixture was added into each panel, and the practical volume was approximately 654.5 nL (0.85 nL × 770), whereas the rest of the reaction was the dead volume.

The operational step of the digital array contains four steps: priming the chips, transferring the reaction mixture, loading the chip and amplification. The final 4-μL reaction volume of each digital panel contains 1× Taqman Gene Expression Mix with a positive ROX fluoresce signal (Life Technologies), 2× sample loading reagent (Fluidigm), forward and reverse event-specific and endogenous primers at a final concentration of 225 and 50 nM of the probes, 50 ng of undigested DNA template, and additional ddH_2_O to make up the remainder of the 4 µL. In the above reaction volume, as the predicted copy number of each endogenous gene is 18,315 copies per microliter, which is based on the theoretical mass of the *zea mays* genome of 2.73 pg [35]. Three parallel reactions were set up in our experiment, and all the experimental data were collected and analyzed for our final conclusion.

#### 4.6.2. The Droplet Digital PCR

In our assay, the droplet digital PCR was performed using RainDrop^TM^ digital PCR system (RainDance Technologies Inc., Billerica, MA, USA) and Bio-Rad QX100 droplet system (Bio-Rad, Pleasanton, CA, USA).

This ddPCR platform of RainDrop contains three steps: the generation of the oil-water structure, the PCR amplification and the droplet detection by the cytometer. The final volume for the ddPCR was 30 µL, which contained 1× Taqman Gene Expression Mix, 225 and 50 nM of the primers and probes, respectively, 1× droplet stabilizer (RainDance Technologies Inc.), 200 ng of the template DNA (either digested or undigested) and additional ddH_2_O to make up the remainder of the 30 µL.

The ddPCR reaction volume for Bio-Rad included 1× ddPCR Master Mix (Bio-Rad), 225 nM of the forward and reverse primers, 50 nM of each probe at the same concentration used for optimized cdPCR, and 250 ng DNA template, with ddH_2_O added to bring the final volume to 20 µL.

The thermal cycling conditions for both the cdPCR and the ddPCR were set up as follows: 50 °C for 5 min, 95 °C for 5 min, and 50 cycles of 95 °C for 1 min and 60 °C for 1 min, during which the fluoresce signal was collected.

### 4.7. Data Analysis

The BioMark software (Fluidigm, South San Francisco, CA, USA) can generate PCR amplification curves and analyze the threshold values of the 36,960 individual partitions (48 × 770). Following the amplification, the amplification curves were analyzed automatically using the Digital PCR Analysis Software (Fluidigm). The wells that achieved *C*t values between 25 and 45 were regarded as the positive partitions.

The detection data of ddPCR of RainDrop and Bio-Rad platforms were analyzed by the software of RainDrop analyst (RainDance Technologies Inc.) and Bio-Rad analysis (Bio-Rad), respectively. The threshold of each reaction was set up manually for distinguishing the negative and positive signals.

We counted the number of positive partitions for both the event-specific and endogenous amplifications for each sample. According to the Poisson distribution, the original copy number of the different samples can be calculated based on the following equations [36]:

(1) A(endogenous gene)=−ln [(N−X)/N]×N

(2) B(event−specific gene)=−ln [(N−Y)/N]×N

In the above two equations, *A*(endogenous gene) and *B*(event-specific gene) were the estimated copy number of the endogenous and event-specific genes for each panel, respectively; *N* was the total number of partitions; and X and Y were the positive wells for the endogenous and event-specific genes, respectively. Finally, the GMO content can be calculated using the following equation:

(3) $$\text{GMO content }(\%)=\frac{A\left(\text{event}-\text{specific gene}\right)}{B\left(\text{endogenous gene}\right)}\times 100\%$$

### 4.8. Real-Time PCR Analysis

The real-time PCR experiments were carried out using a Bio-Rad C1000 real-time PCR platform (Bio-Rad). The concentration of each PCR reaction was in accordance with the concentration of the duplex cdPCR, in addition to the thermal cycling conditions. The data were analyzed according to a previous method [15].

## 5. Conclusions

An absolute quantitative detection system based on the pretreatment-free duplex chamber digital PCR was developed in this article. This detection system could achieve quantitative detection for the GMO genomic sample with target template as low as 0.5%, and qualitative detection above 0.1%, lower than the common qPCR. This could meet all the demands of the labeling law of different countries and groups. Moreover, according to the evaluation of influence factors of this system, the digestion of genome sample and the SNP sites were proved the importance to the final detection result. This provided this detection system as an SNP detection method with bright potential.

## Figures and Tables

**Table 1:** The sensitivity detection for both the limit of quantitation (LOQ) and the limit of detection (LOD).

Mass Concentration ^a^	GA21 Event-Specific Gene ^b^				Endogenous Gene				Copy Number Concentration ^d^			
	1 ^c^	2	3	RSD	1	2	3	RSD	1	2	3	RSD
100%	261	282	282	3.60%	617	635	617	1.36%	42.3%	44.4%	45.7%	3.17%
50%	140	122	150	8.44%	565	563	601	3.03%	24.8%	21.7%	25%	6.34%
20%	45	50	44	5.66%	505	497	501	0.65%	8.9%	10.1%	8.8%	6.37%
5%	16	17	14	7.96%	511	491	509	1.79%	3.1%	3.4%	2.7%	9.35%
1%	3	4	3	14.14%	505	480	514	2.88%	0.6%	0.8%	0.5%	19.69%
0.5%	2	2	1	24.28%	597	524	532	5.93%	0.3%	0.4%	0.3%	24.22%
0.1%	1	0	1	–	588	556	571	2.80%	0.3%	0	0.3%	–
Negative	0	0	0	–	600	589	591	0.99%	0	0	0	–

^a^ In this table, the first row was defined as the “GMO concentration determined by mass percentage”, on the other word, this concentration was calculated by the ratio of GMO mass and the total mass; ^b^ In this table, the row of “GA21 Event-specific Gene” was defined as the copy number of GA21 event that detected for the each panel of each sample. The copy number was according to the reaction volume of the chamber-based digital PCR. The row of “Endogenous Gene” is as same as this; ^c^ The number of “1,2,3” was defined as the three parallels in our experiments for each detection sample; ^d^ The “Copy Number Concentration” was defined as the practical concentration that based on the ratio of the copy number of event-specific and reference gene. The values were calculated based on the Equation (3). RSD: relative standard deviation.

**Table 2:** The result of droplet digital polymerase chain reaction achieved by Raindrop and Bio-Rad system.

Entry	Total Droplets	Pretreatments Including						Pretreatment-Free					
		Copy Number of Endogene ^1^		Copy Number of Event-Specific Gene		The GMO Content by Copy Number		Copy Number of Endogene		Copy Number of Event-Specific Gene		The GMO Content by Copy Number	
RainDrop	5,000,000	5825	5136	2281	2016	39.2%	39.3%	7939	7099	3493	3220	44.0%	45.4%
Bio-Rad	20,000	5937	5648	2333	2236	39.3%	39.6%	7815	7449	3430	3232	43.9%	43.4%

^1^ All the copy numbers in this sheet as the copy number of each target gene with the final volume of 20 µL.

**Table 3:** The ΔG and non-specific hybridization sites between the probes and unspecific primers.

Primer Name	Sequence of Duplex Probe	The Hybridization Part	The Hybridization ΔG ^1^
GA21-P-4	AATCAGGGCTCATTTTCTCGCTCCTCA	….GAGA….-3′….CTCG….-5′	−6.31 kcal/mol
GA21-P-5		….GAGCC….-3′….CTCGG….-5′	−9.38 kcal/mol
A135-P-4	TTTCTCAACAGCAGGTGGGTCCGGGT	….GCTG….-3′….CGAC….-5′	−6.69 kcal/mol
A135-P-5		….TGCTG….-3′….ACGAC….-5′	−8.65 kcal/mol

^1^ For the ΔG in this sheet, the values were determined at the PCR condition, that 60 °C for the temperature and the iron concentration of the MasterMix.

**Table 4:** The primers and probes for our study.

Target Genes	Primers/Probes Name	Primers/Probes Sequence	Reference
Adh1 reference gene	Adh1-70F	5′-CCTTCTTGGCGGCTTATCTG-3′	[33]
	Adh1-70R	5′-CCAGCCTCATGGCCAAAG-3′	
	Adh1-70P	5′-VIC-CTTAGGGGCAGACTCCCGTGTTCCCT-BHQ1-3′	
	Adh1-135F	5′- CGTCGTTTCCCATCTCTTCCTCC-3′	[34]
	Adh1-135R	5′-CCACTCCGAGACCCTCAGTC-3′	
	Adh1-135P	5′-VIC-AATCAGGGCTCATTTTCTCGCTCCTCA-BHQ1-3′	
GA21 event-specific gene	GA21-F	5′-CGTTATGCTATTTGCAACTTTAGAACA-3′	[34]
	GA21-R	5′-GCGATCCTCCTCGCGTT-3′	
	GA21-P	5′-FAM-TTTCTCAACAGCAGGTGGGTCCGGGT-BHQ1-3′	
Primers for unspecific hybridization	GA21-P-4	5′-TTTCTCAA**GAGC**AGGTGGGTCCGGGT-3′	This research
	GA21-P-5	5′-TTTCTCAA**GAGCC**GGTGGGTCCGGGT-3′	
	A135-P-4	5′-AATCAGG**GCTG**ATTTTCTCGCTCCTCA-3′	
	A135-P-5	5′-AATCAG**TGCTG**ATTTTCTCGCTCCTCA-3′	
Primers for SNP researches ^2^	A70-R-M-A-G	5′-CCAGCCTCGTGGCCAAAG-3′	This research
	A135-F-M-A-C	5′-CGTCGTTTCCCCTCTCTTCCTCC-3′	
	A135-F-M-A-G	5′-CGTCGTTTCCCGTCTCTTCCTCC-3′	
	A135-F-M-A-T	5′-CGTCGTTTCCCTTCTCTTCCTCC-3′	
	A135-F-3′-C-A	5′-CGTCGTTTCCCATCTCTTCCTCA-3′	
	A135-F-3′-C-T	5′-CGTCGTTTCCCATCTCTTCCTCT-3′	
	A135-F-3′-C-G	5′-CGTCGTTTCCCATCTCTTCCTCG-3′	
	A135-F-5′-C-G	5′-GGTCGTTTCCCATCTCTTCCTCC-3′	
	A135-F-5′-C-A	5′-AGTCGTTTCCCATCTCTTCCTCC-3′	
	A135-F-5′-C-T	5′-TGTCGTTTCCCATCTCTTCCTCC-3′	

^1^ For the primers of the research of unspecific hybridization, the bases in bold style means the unspecific hybridization bases to the probes within the duplex detection; ^2^ For the primers of the SNP researches, the bases with an underline means the SNP sites compared to the original sequence.

## Supplementary Materials

Supplementary materials can be found at http://www.mdpi.com/1422-0067/17/3/402/s1.

## Abbreviations

The following abbreviations are used in this manuscript:

PCR: polymerase chain reaction
GMO: genetically modified organism
SNP: single nucleotide polymorphisms
LOD: limit of detection
LOQ: limit of quantitation
