# Supplementary material for: A Novel Pretreatment-Free Duplex Chamber Digital PCR Detection System for the Absolute Quantitation of GMO Samples
Source: Int J Mol Sci. 2016 Mar 18;17(3):402. doi: 10.3390/ijms17030402 (PMC4813257; doi:10.3390/ijms17030402)
Supplement: Supplementary file 1 [file ijms-17-00402-s001.pdf]

Int. J. Mol. Sci. **2016**, *17*, 402; doi:10.3390/ijms17030402

Supplementary Materials: A Novel Pretreatment-Free Duplex Chamber Digital PCR Detection System for the Absolute Quantitation of GMO Samples

Pengyu Zhu, Chenguang Wang, Kunlun Huang, Yunbo Luo and Wentao Xu

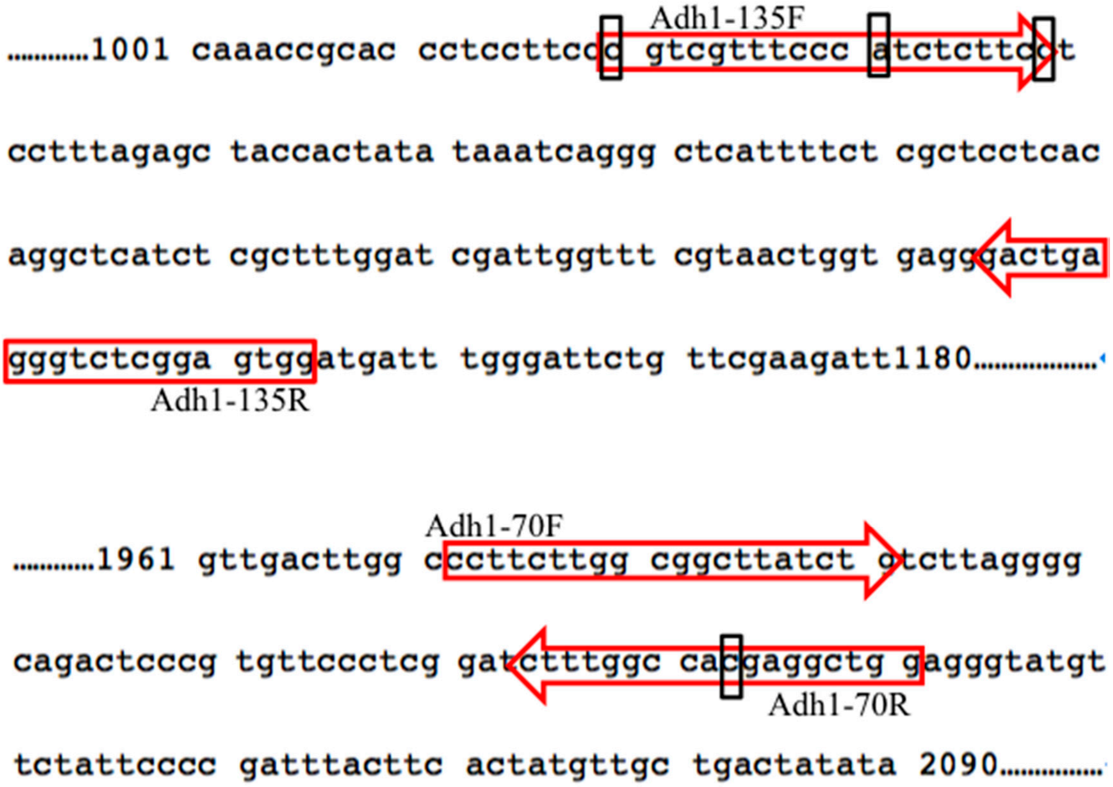

Figure S1. The location of the primers in the Adh1 gene. The arrows in red mean the sequences and directions of primers. The columns in black mean the SNP sites evaluated in our study.

Table S1. The real-time PCR results for the evaluation of nonspecific annealing bases.

Probe Name	Nonspecific Annealing Primer	The Bases of Nonspecific Annealing	The Amplification Efficiency	The R^2 of Standard Curve
Adh1-135P	None	0	85.6%	0.990
	GA21-P-4	4	93.1%	0.988
	GA21-P-5	5	89.2%	0.997
GA21-P	None	0	91.5%	0.997
	A135-P-4	4	90.8%	0.996
	A135-P-5	5	92.2%	0.995
Duplex assays	None	None	83.6% (For GA21)	0.987 (For GA21)
	None	None	86.7% (For A135)	0.992 (For A135)

Table S2. The real-time PCR results for the evaluation of SNP site of Adh1-70R.

The Primer Assays	The Exist of SNP Site	The Amplification Efficiency	The R^2 of Standard Curve	The Equation of Standard Curve
Adh1-70F/Adh1-70R	One SNP in the Adh1-70R	86.6%	0.967	$Ct = -3.722 lgC + 30.371$
Adh1-70F/A70-R-M-A-G	No SNP sites	102.4%	0.997	$Ct = -3.265 lgC + 33.173$
